# Plant Cell Wall Dynamics in Compatible and Incompatible Potato Response to Infection Caused by *Potato Virus Y* (PVY^NTN^)

**DOI:** 10.3390/ijms19030862

**Published:** 2018-03-15

**Authors:** Katarzyna Otulak-Kozieł, Edmund Kozieł, Benham E. L. Lockhart

**Affiliations:** 1 Department of Botany, Faculty of Agriculture and Biology, Warsaw University of Life Sciences—SGGW, 159 Nowoursynowska St., 02-776 Warsaw, Poland; edmund_koziel@sggw.pl; 2 Department of Plant Pathology, University of Minnesota, St. Paul, MN 55108, USA; lockh002@umn.edu

**Keywords:** cell wall, cellulose synthase, hypersensitive response, pathogenesis related-protein 2, plant-virus interaction, *Potato virus Y*, ultrastructure

## Abstract

The cell wall provides the structure of the plant, and also acts as a barier against biotic stress. The vein necrosis strain of *Potato virus Y* (PVY^NTN^) induces necrotic disease symptoms that affect both plant growth and yield. Virus infection triggers a number of inducible basal defense responses, including defense proteins, especially those involved in cell wall metabolism. This study investigates the comparison of cell wall host dynamics induced in a compatible (potato cv. Irys) and incompatible (potato cv. Sárpo Mira with hypersensitive reaction gene *Ny-Smira*) PVY^NTN^–host–plant interaction. Ultrastructural analyses revealed numerous cell wall changes induced by virus infection. Furthermore, the localization of essential defensive wall-associated proteins in susceptible and resistant potato host to PVY^NTN^ infection were investigated. The data revealed a higher level of detection of pathogenesis-related protein 2 (PR-2) in a compatible compared to an incompatible (HR) interaction. Immunofluorescence analyses indicated that hydroxyproline-rich glycoproteins (HRGP) (extensin) synthesis was induced, whereas that of cellulose synthase catalytic subunits (CesA4) decreased as a result of PVY^NTN^ infection. The highest level of extensin localization was found in HR potato plants. Proteins involved in cell wall metabolism play a crucial role in the interaction because they affect the spread of the virus. Analysis of CesA4, PR-2 and HRGP deposition within the apoplast and symplast confirmed the active trafficking of these proteins as a step-in potato cell wall remodeling in response to PVY^NTN^ infection. Therefore, cell wall reorganization may be regarded as an element of “signWALLing”—involving apoplast and symplast activation as a specific response to viruses.

## 1. Introduction

The cell wall serves both to define and maintain the structural integrity of the plant as well as to protect from external stress. It is estimated that as much as 15% of the plant genome is associated with cell wall metabolism and biosynthesis. It also functions to adapt the plant to changes during growth and development [1]. This involves modifications in composition and structure of the cell wall through sensing and signaling, intercellular communication as well as exchange interfaces [2,3].

*Potato virus Y* (genus *Potyvirus*) has been listed as the fifth most economically damaging plant virus in the world [4]. The symptoms of PVY infection depend on virus strain and host resistance level. It infects more than 170 species belonging to 34 genera and has a worldwide distribution. The PVY^NTN^, or vein necrosis strain, induces diverse symptoms including necrotic ringspotting which greatly decreases the market quality of harvested tubers [5]. Host response to virus infection is categorized as either compatible (susceptible) or incompatible (resistant) interactions. In the former, the virus spreads systemically throughout the host, whereas in the latter, systemic spread is prevented by localized cell death (hypersensitive response, HR) [6,7].

Viruses, like many other pathogens, trigger a number of inducible defense responses when recognized by plants, inducing the up-regulation of a number of common defensive proteins. Proteins/enzymes involved in cell wall metabolism play a crucial role in this interaction through the influence on cell-to-cell virus spread [3]. Following initial infection, viruses are potentially able to invade adjacent cells, but systemic spread depends on the mechanism involved in cell-to-cell movement [8]. The cell wall may act as a physical barrier to virus infection as well as to restrict intercellular movement [9]. Initial infection of the plant occurs via mechanical wounding or insect feeding that breeches the surface (epidermal) layer [10]. Following replication in initial sites of infection, both intra-cellular and inter-cellular movement of progeny virus depends on the intervention of several host mechanisms, including plasmodesmata modifications [11]. Current information on modifications in composition, structure and function of plant cell walls as related to defense against pathogen invasion has been largely restricted to non-viral pathogens [12,13,14]. This work has concentrated mainly on pectin, pectic polysaccharide or glycan modifications during plant-microbe interaction, and has focused on cell wall polymers such as callose [15] or lignin [16]. However, knowledge about crucial components in the active cell wall defense strategies relative to responses to other pathogens is lacking. Recent proteomic studies have suggested that certain proteins undergo regulation during plant virus infection. This, as well as other studies on molecular host-virus proteins interaction, suggest that changes in cell wall-associated proteins and cell wall metabolism may play an important function in regulating cell-to-cell trafficking of plant viruses [7,17]. Therefore, this study focuses on ultrastructural cell wall changes and dynamics in potential rearrangements of cell wall proteins in response to PVY^NTN^ infection using differences between susceptible and hypersensitive resistant potato varieties. This study describes for the first time the precise ultrastructural changes occurring in the two types of PVY^NTN^-potato interactions (compatible and incompatible). The findings reveal changes in the regulation of protein production involved in potato cell wall synthesis (e.g., CesA4), and localization of proteins arranged in cell wall remodeling processes (pathogenesis related protein (PR-2) and HRGP (extensin)).

## 2. Results

### 2.1. Ultrastructural Analyses of Adjoining Cell Wall Connections in Compatible and Incompatible PVY^NTN^-Potato Interactions

**Figure 1:**
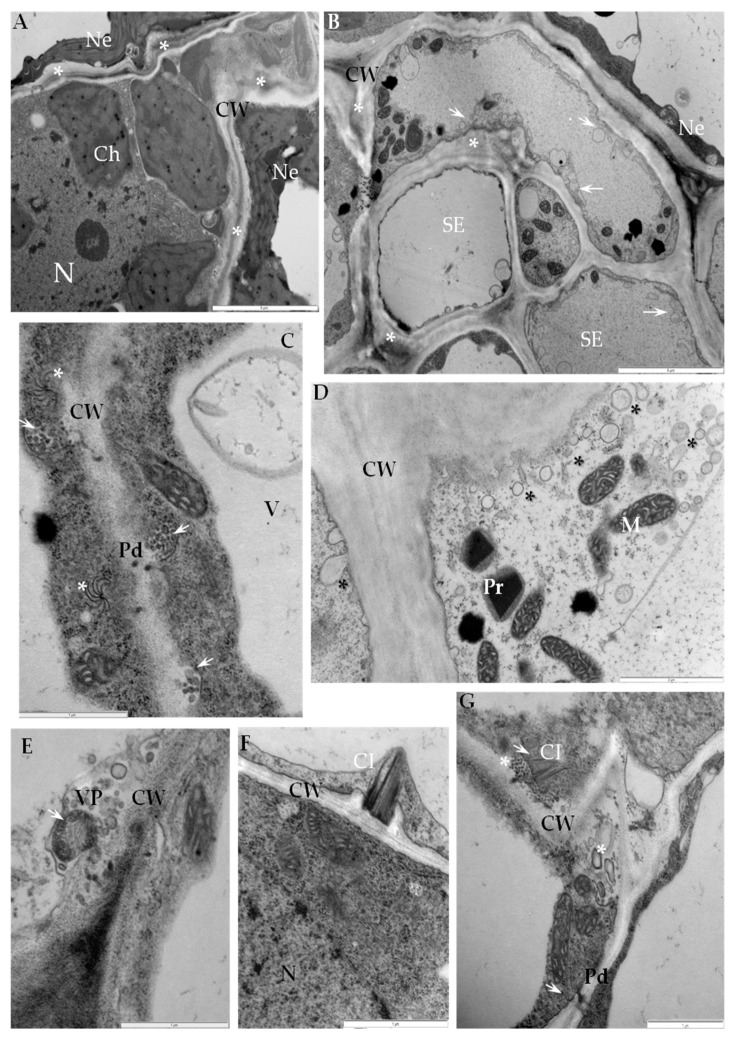
Ultrastructure changes in cell wall area from potato Irys–PVY^NTN^ interaction. (**A**) Cell wall loosening and folding (*) between necrotized and non-necrotized mesophyll cells. Bar 5 µm. (**B**) Cell wall thickening (*) with paramural bodies (arrows) in phloem cells. Bar 5 µm. (**C**) Paramural bodies (arrow) connected with plasmodesmata in association with virus cytoplasmic inclusions (*). Bar 1 µm. (**D**) Cell wall thickening with intense vesicles distribution (*). Bar 2 µm. (**E**) PVY particles (arrow) in vesicle derived from cell wall with other paramural bodies in the vicinity of changed cell wall. Bar 1 µm. (**F**) PVY inclusions in associated with highly changed cell wall in mesophyll cell. Bar 1 µm. (**G**) PVY inclusions (arrow) associated with plasma membranes and intense distribution of paramural bodies (asterisk) along the deformed cell wall. Bar 1 µm. Ch—chloroplast, CI—cytoplasmic inclusions, CW—cell wall, ER—endoplasmic reticulum, N—nucleus, Ne—necrosis, Pd—plasmodesmata, SE—sieve element, V—vacuole, VP—virus particles.

Changes in cell wall ultrastructure induced by PVY^NTN^ infection were analyzed ten days after mechanical inoculation of potato cvs. Irys (compatible) and Sárpo Mira (hypersensitive). In the susceptible potato-PVY^NTN^ interaction the cell wall folding with loosening structure was mainly observed in mesophyll cells and also in necrotic areas (Figure 1A). In compatible interactions multivesicular bodies connected with plasmodesmata were noticed in associations with PVY^NTN^ cytoplasmic inclusions (Figure 1C). Moreover, modifications of cell wall area were very strongly associated with membrane and vesicular structures and also with presence of PVY particles or inclusions (Figure 1E). Additionally, in mesophyll or parenchyma in the vascular bundle area, complexes of viral cytoplasmic inclusions and plasmodesmata resulted in cell wall protrusions (Figure 1F,G). Phloem tissue typically showed abnormal thickening of cell walls with numerous plasma membrane-derived vesicles (Figure 1B,D).

**Figure 2:**
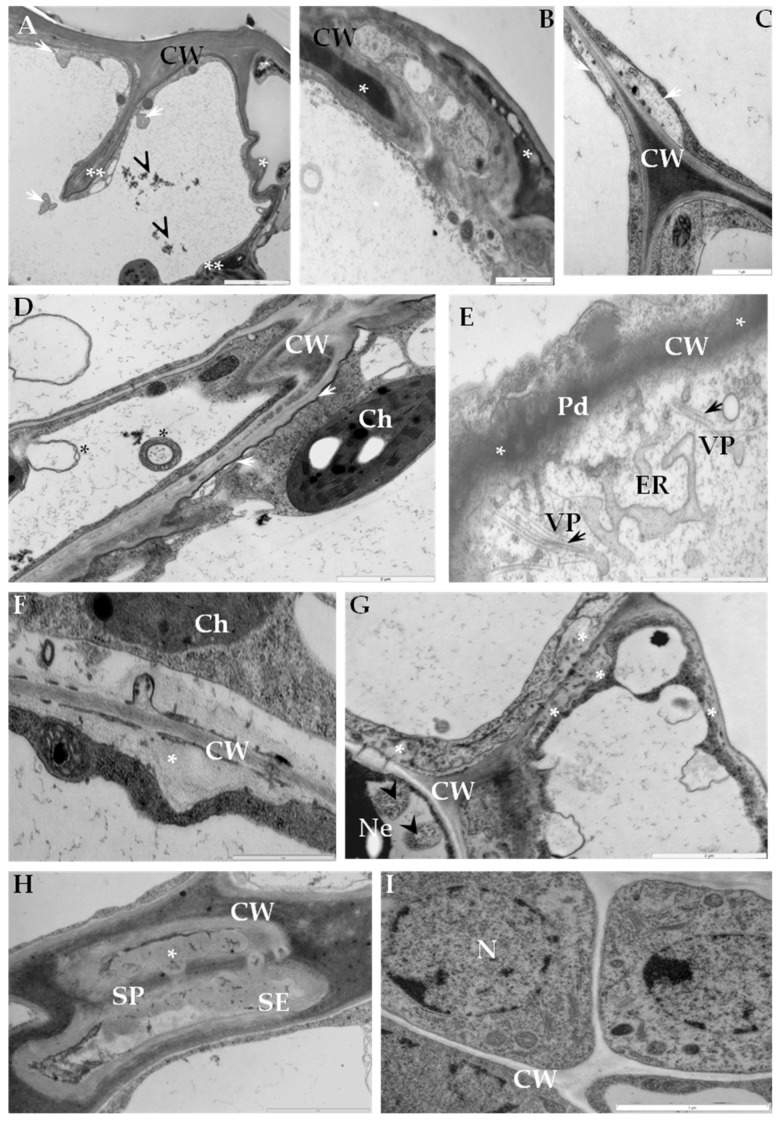
Ultrastructure changes in cell wall area from potato Sárpo Mira–PVY^NTN^ interaction. (**A**) The cell wall folding (*) and invagination (**) in epidermis during HR. Multivesicular bodies (arrows) and phenolic compounds (arrowhead) presented in vacuole. Bar 2 µm. (**B**) Electron dense phenolic compounds material in reinforced epidermis cell wall (*). Bar 1 µm. (**C**) Plasma membrane retrations from cell wall (arrows) between mesophyll cells. Bar 1 µm. (**D**) Reinforced cell wall between mesophyll cells, phenolic compounds as a layer between plasma membrane and cell wall (arrows). Multivesicular bodies in vacuole (*). Bar 2 µm. (**E**) Expanded plasmodesmata area, enlarged endoplasmic reticulum, virus particles associated with membranous structure of ER (arrows). Phenolic compounds as an electron dense cell wall (asterisk). Bar 2 µm. (**F**) Callose material deposition between cell wall and plasma membrane in mesophyll cells. Bar 1 µm. (**G**) Callose deposition (asterisk) between phloem parenchyma cells. Multivesicular bodies in vacuole of necrotized cell (arrowhead). Bar 2 µm. (**H**) Callose (*) around sieve plate in sieve element. Bar 2 µm. (**I**) Phloem parenchyma cells from mock-inoculated leaf (control material). Bar 1 µm. Ch—chloroplast, CW—cell wall, ER—endoplasmic reticulum, Pd—plasmodesmata, SE—sieve element, V—vacuole, VP—virus particles.

In incompatible interactions extensive disruptions of epidermal cell wall structure were observed (Figure 2A,B). In sections of mesophyll, the plasma membrane was separated from cell wall (Figure 2C). Irregular thickening and folding of the cell wall was also observed in the hypersensitive response (Figure 2D). Phenolic-like compounds were observed as electron dense areas within cell walls (Figure 2E) or as layers between cell walls and plasmalemma (Figure 2D). Plasmodesmata enlargement in the vicinity of enlarged endoplasmic reticulum membranes were observed primarily in mesophyll tissue, but virus particles were rarely observed (Figure 2E). The deposition of callose-like material was associated with phloem adjacent cells, especially in sieve elements (Figure 2E–H). Multivesicular bodies were detected in phloem tissue and observed changes indicated clearly that cell wall material was being translocated to the vacuoles (Figure 2G). Neither cell wall disruption, nor virus particles and inclusions were observed in comparable healthy tissues (mock-inoculated) (Figure 2I). 

### 2.2. Localization of Pathogenesis Related Protein 2 (PR-2, β-1,3 Glucanase, EC 3.2.1.39)

**Figure 3:**
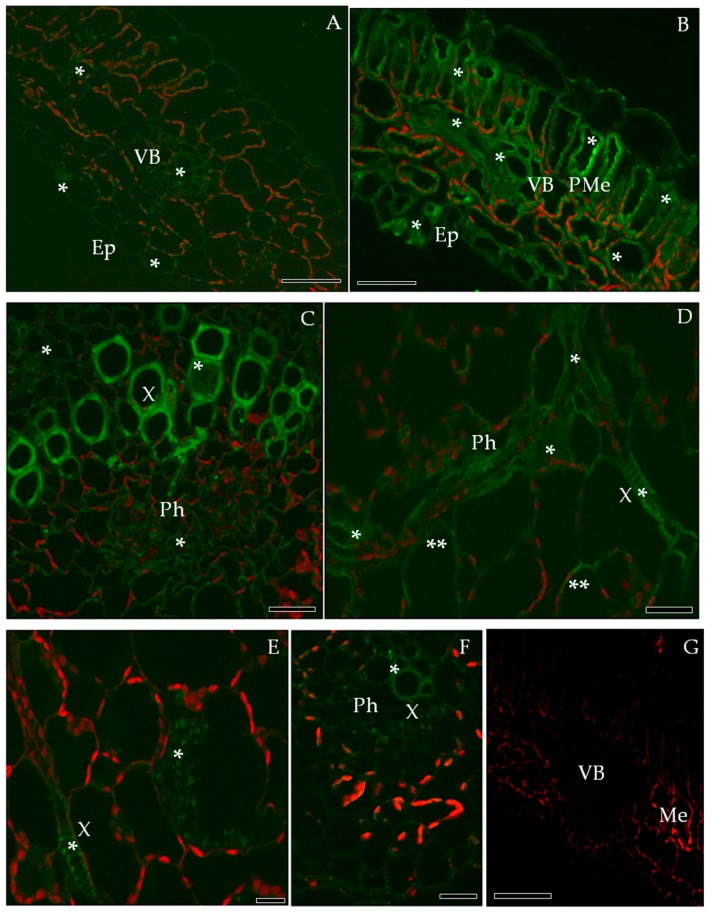
Immunofluorescence localization of PR-2 protein in potato-PVY^NTN^ compatible (**B**,**C**) and incompatible interaction (**D**–**G**). (**A**) Green fluorescence signal of PR-2 in vascular bundles and epidermis (asterisk) of mock-inoculated potato leaf (control). Bar 200 µm. (**B**) Green fluorescence of PR-2 in palisade mesophyll, vascular bundle and epidermis with stomata (asterisk) of cv. Irys infected with PVY^NTN^. (**C**) Fluorescence PR-2 signal in xylem and phloem elements (asterisk) of potato Irys infected with PVY^NTN^. (**D**) PR-2 signal in phloem & xylem element (*) of Sárpo Mira inoculated with PVY^NTN^, also in the cell wall of spongy parenchyma cells (**). (**E**) PR-2 in the cell wall of xylem elements and in the symplast of spongy mesophyll. (**F**) PR-2 in the cell wall of phloem and xylem elements inside Sárpo Mira leaflets. Bar 200 µm. (**G**) Control—lack of green fluorescence signal in hypersensitive response when primary antibodies were omitted. Bar 200 µm. Ep—epidermis, Me—mesophyll, Ph—phloem, PMe—palisade mesophyll, VB—vascular bundle, X—xylem.

**Figure 4:**
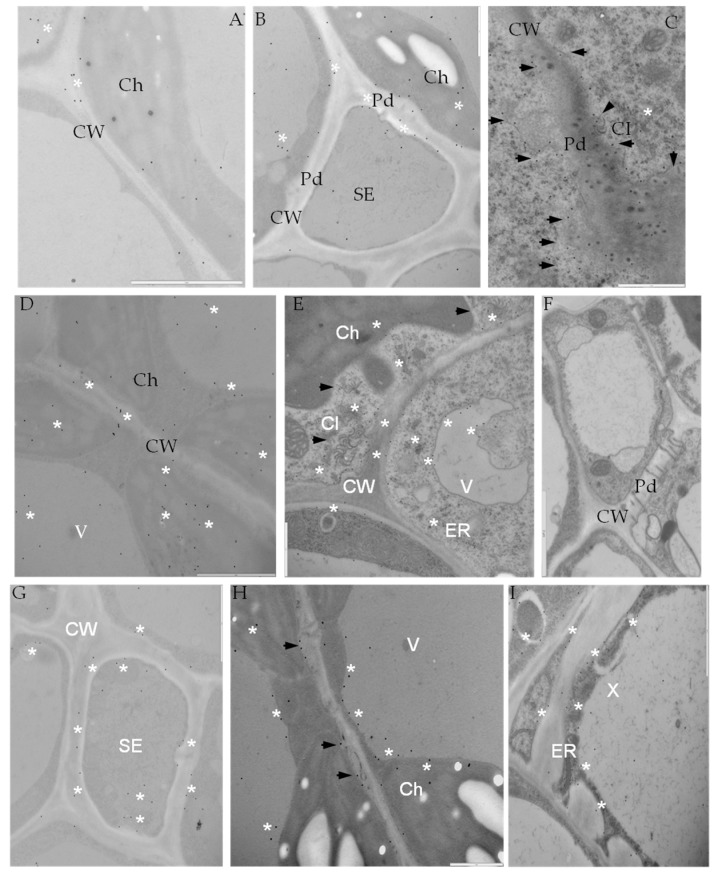
Immunogold labeling of PR-2 in potato-PVY^NTN^ compatible (**B**–**E**) and incompatible interaction (**F**–**I**). (**A**) Gold deposition in mesophyll of mock-inoculated leaf. Bar 2µm. (**B**) PR-2 deposition in sieve elements in potato Irys inoculated with PVY^NTN^. Bar 1 µm. (**C**) PR-2 deposition in plasmodesmata area and around (*) membranous structure (arrows) in phloem parenchyma. Virus inclusions associated with plasmodesmata. Bar 1 µm. (**D**) PR-2 localization (*) in symplast of potato Irys mesophyll cells. Deposition in cell wall in plasmodesmata area, chloroplast and vacuole. Bar 2 µm. (**E**) PR-2 localization along membranous structures (asterisk) [ER, tonoplast and plasmalema]. Virus inclusion associated to membranes (arrows). Bar 1 µm. (**F**) Control—lack of gold deposition in hypersensitive response when primary antibodies were omitted. Bar 2 µm. (**G**) PR-2 location (*) in sieve elements in HR response. Bar 1 µm. (**H**) PR-2 location along expanded cell wall area in the vicinity of plasmodesmata (arrows). Localization also in vacuole and chloroplasts (asterisk). Bar 1 µm. (**I**) PR-2 deposition inside xylem tracheary elements, along cell wall and along ER or in vacuole. Bar 1 µm. Ch—chloroplast, CI—cytoplasmic inclusions, CW—cell wall, ER—endoplasmic reticulum, Pd—plasmodesmata, SE—sieve element, V—vacuole, X—xylem tracheary element.

The immunofluorescence localization of PR-2 in susceptible and resistant potato leaves indicated that PR-2 was induced by PVY^NTN^ infection (Figure 3), but absent in controls (Figure 3A,G). Green fluorescence due to PR-2 accumulation was noticeably higher in the compatible interaction (Figure 3B,C), as compared to the hypersensitive response (Figure 3D–F). In cv. Irys leaf tissues green signal developing in mesophyll cells, stomata and vascular bundles, was higher in inoculated as compared to mock-inoculated treatments, especially in xylem (Figure 3B,C). In contrast, PR-2 deposition associated with hypersensitive response occurred mainly in xylem and phloem elements (Figure 3D–F) with scattered symplast deposition in spongy mesophyll cells adjacent to the vasculature (Figure 3E,F). Immunogold labeling revealed that PR-2 occurred in areas of the cell wall (Figure 4B–E,G–I), often adjacent to plasmodesmata (Figure 4B–E,G–H) and also associated with presence of virus inclusions. PR-2 was detected attached to the symplast membranous structure in phloem parenchyma (Figure 4C) and mesophyll cells (Figure 4E,H).

**Figure 5:**
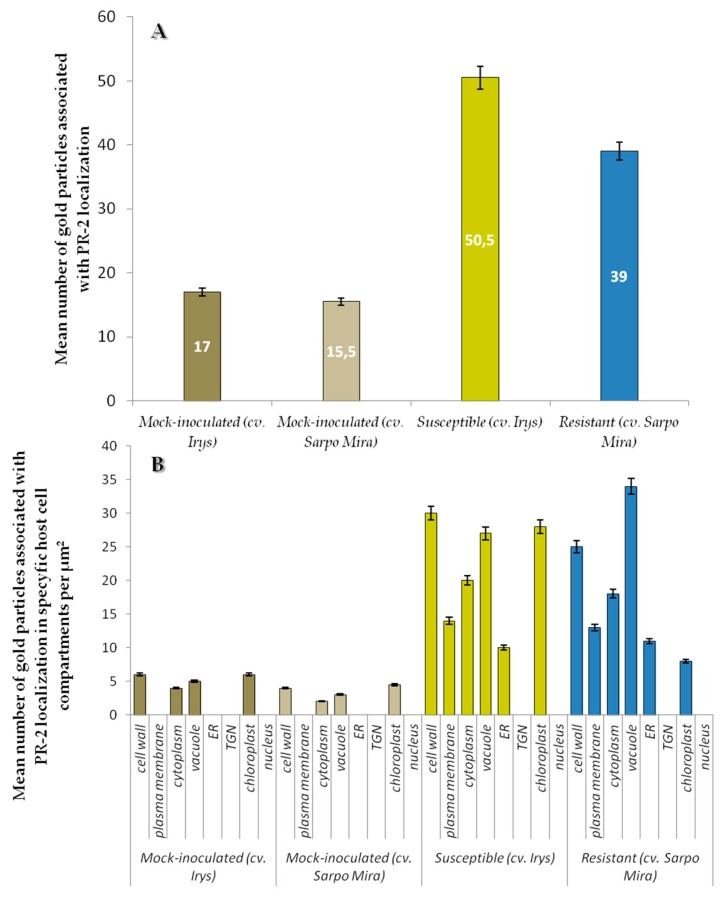
Statistical significance assessment of PR-2 epitopes immunogold localization (**A**) in mock-inoculated and PVY inoculated potato tissues of cultivars Irys (susceptible) and Sárpo Mira (resistant) & (**B**) in specific host cell compartments of mock-inoculated and PVY infected potato plants (susceptible and resistant). Figure presents mean numbers of gold particles located in specific compartment per µm^2^. Quantification immunogold localization was prepared using ANOVA method. Mean values of gold particle localization were evaluated at the *p* < 0.05 level of significance using post-hoc Tukey HSD test.

Levels of PR-2 antigen detection were slightly lower in mock-inoculated cv. Sárpo Mira than in cv. Irys plants (Figure 5A). Regardless of cultivar, PVY infection caused increase of PR-2 presence in host tissues in comparison to controls (Figure 4A–I and Figure 5A). Increase of PR-2 levels in inoculated relative to mock-inoculated controls was 66.3% for susceptible and 60.3% for resistant cultivars. The highest levels of PR-2 was observed in PVY^NTN^ infected potato plants cv. Irys (mean number of gold particles = 50.5) (Figure 5A). A lower rate of PR-2 localization occurred in PVY^NTN^ resistant cv. Sárpo Mira (mean number of gold particles = 39). The difference between susceptible and resistant potato plants was statistically significant and was approximately 23%. The above results suggest that PR-2 protein is present at higher levels during PVY infection in susceptible than in resistant cultivars exhibiting a hypersensitive response. Quantification of PR-2 antigen by immunogold localization in host cell compartments (Figure 5B) indicated that the localization pattern was in general similar to overall tissue localization of PR-2 (Figure 5A). A lack of localization in trans-Golgi network (TGN) was observed. The highest levels of PR-2 localization were noticed in the susceptible PVY-infected cultivar and was greatest in cell walls and the lowest in cytoplasm (Figure 5B). In contrast, in the resistant potato cultivar the level of detectable PR-2 was highest in the vacuole. Levels of PR-2 in the vacuole in plants undergoing a hypersensitive response exceeded not only that occurring in mock-inoculated plants, but also in cv. Irys plants systemically infected with PVY. Immunogold labeling results showed induction of PR-2 in all cell compartments of PVY-infected potato cultivars cv. Irys and cv. Sárpo Mira compared to mock-inoculated plants. Statistical analyses of PR-2 localization also indicated different pattern of plant reaction to PVY^NTN^ infection depending on the level of resistance. In susceptible cultivars during PVY infection, PR-2 was primarily located in the cell wall. This specific protein localization is directly associated with host cell wall modification induced by the pathogen to increase virus mobility. Similar cell wall modification was also observed by ultrastructural analysis. A different host–plant response was observed in PVY-infected resistant plants, in which PR-2 was strongly sequestered in the vacuole, and in this case storage of PR-2 was seldom observed in the cell wall. During the HR response induced by PVY, PR-2 is likely actively withdrawn or stored in the vacuole.

### 2.3. Localization of Cellulose Synthase 4 (CesA4, EC 2.4.1.12)

**Figure 6:**
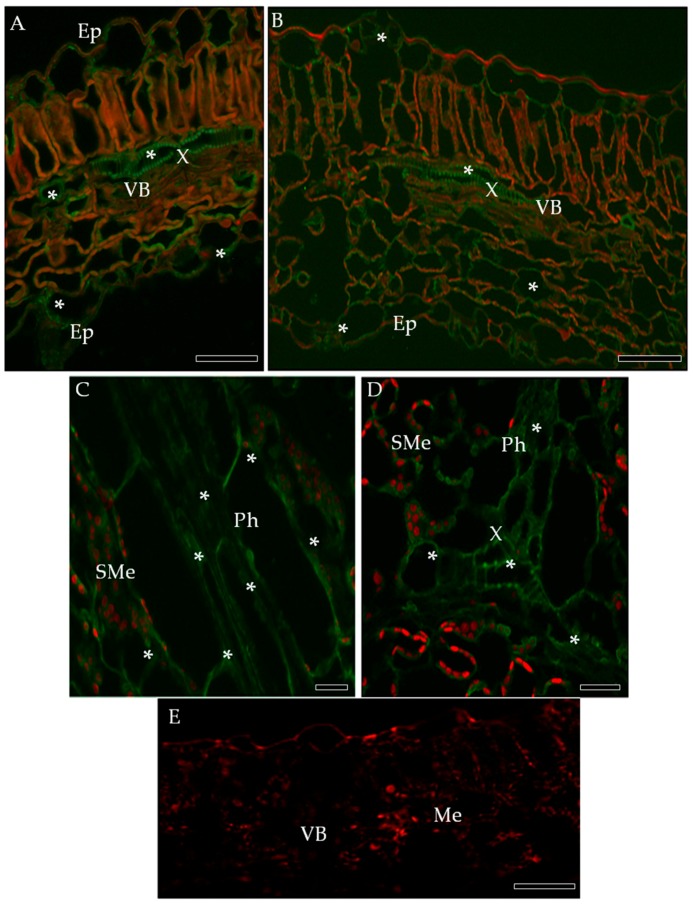
Fluorescence detection of CesA4 protein in potato–PVY^NTN^ compatible (**B**) and incompatible interaction (**C**–**E**). (**A**) CesA4 signal (*) in xylem elements and epidermis of mock-inoculated leaf. (**B**) CesA4 signal (*) in xylem, spongy mesophyll cell wall and epidermis (also in stomata) of Irys leaf inoculated with PVY^NTN^. (**C**) CesA4 green fluorescence in cell wall and symplast of phloem elements in Sárpo Mira leaf (asterisk). Signal also in spongy mesophyll cell wall. (**D**) CesA 4 (*) detection in spongy mesophyll and vasculature of Sárpo Mira. (**E**) Control-lack of green fluorescence in hypersensitive response when primary antibodies were omitted. Bar 200 µm. Ep—epidermis, Ph—phloem, SMe—spongy mesophyll, VB—vascular bundle, X—xylem.

Immunofluorescence detection of CesA4 antigen gave similar results in both susceptible and resistant potato cultivars (Figure 6B–D) in comparison to controls (Figure 6A,E). CesA4 signal was observed mainly in xylem elements, but also in spongy mesophyll regardless of potato resistance level (Figure 6B–D). In addition, dispersed fluorescence occurred also in epidermis with stomata in the compatible interaction (Figure 6B). The CesA4 signal comes not only from the cell wall, but also from the symplast (Figure 6C).

**Figure 7:**
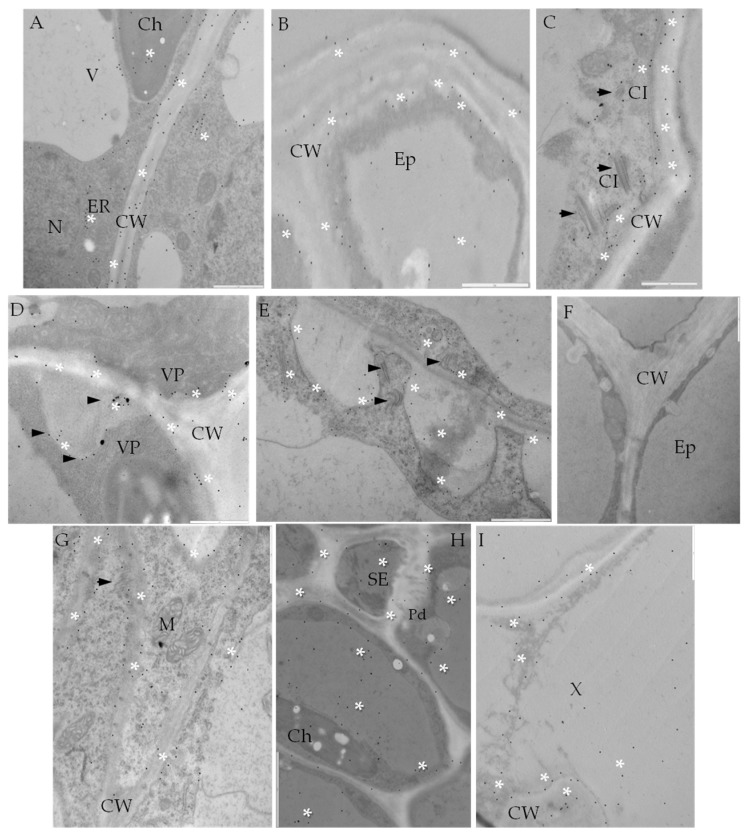
Immunogold labeling of CesA4 in potato–PVY^NTN^ compatible (**B**–**E**) and incompatible interaction (**F**–**I**). (**A**) Gold deposition (*) of CesA4 along cell wall, plasmolema and ER in mesophyll cells of potato mock-inoculated. Bar 1 µm. (**B**) CesA4 deposition (*) in cell wall and vacuole of epidermis cell. Bar 1 µm. (**C**) CesA4 deposition along cell wall and membranous structures associated with virus cytoplasmic inclusions (arrows). Bar 1 µm. (**D**) CesA4 localization (*) along cell wall in potato Irys mesophyll cells. Virus particles around plasmodesmata, gold deposition in area of protoplast retraction from the cell wall (arrow). Bar 1 µm. (**E**) CesA4 localization (*) in xylem tracheary elements. Deposition along cell wall, membranous structures associated with virus inclusions (arrows) and trans Golgi network. Bar 1 µm. (**F**) Control—lack of gold deposition in hypersensitive response when primary antibodies were omitted. Bar 1 µm. (**G**) CesA4 localization (*) along plasma membrane structures and plasmalema (also in connection with cytoskeleton, arrow). Bar 1 µm. (**H**) Gold deposition (*) in cell wall associated with plasmodesmata in sieve element in hypersensitive reaction. Localization also in symplast, especially in vacuole. Bar 2 µm. (**I**) CesA4 localization inside xylem tracheary elements in hypersensitive reaction. Bar 2 µm. Ch—chloroplast, CI—cytoplasmic inclusions, CW—cell wall, Ep—epidermis, ER—endoplasmic reticulum, M—mitochondria, Pd—plasmodesmata, SE—sieve element, V—vacuole, VP—virus particles, X—xylem tracheary element.

Analysis of CesA4 deposition within the apoplast and symplast (Figure 7) confirmed the active trafficking of this protein as a step-in potato cell wall remodeling in response to PVY^NTN^ infection. CesA4 localization was observed in all leaf tissues from epidermis to xylem tracheary elements in susceptible potato, particularly in areas where virus cytoplasmic inclusions or particles were present (Figure 7B–E). In the hypersensitive response, significant CesA4 deposition was observed in both vascular tissues (Figure 7G–I). 

**Figure 8:**
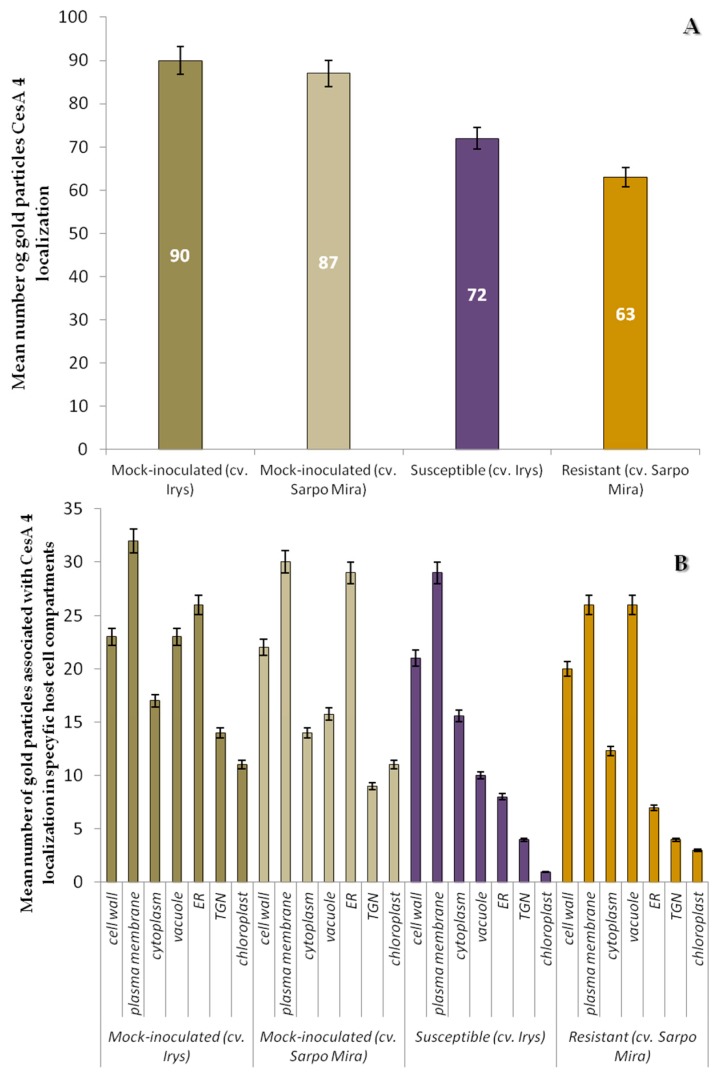
Statistical significance assessment of CesA4 epitopes immunogold localization (**A**) in mock-inoculated and PVY inoculated potato tissues of cultivars Irys (susceptible) and Sárpo Mira (resistant) & (**B**) in specific host cell compartments of mock-inoculated and PVY inoculated potato plants (susceptible and resistant). Figure presents mean numbers of gold particles located in specific compartment per µm^2^. Quantification immunogold localization was prepared using ANOVA method. Mean values of gold particle localization were evaluated at the *p* < 0.05 level of significance using post-hoc Tukey honest significant difference (HSD) test.

The quantification analyses revealed a statistically significant decrease of CesA4 deposition in both types of PVY infected plants, in comparison to controls (Figure 7A,F and Figure 8A). The estimated reduction of CesA4 based on gold particles localization was aproximately 20%-cv. Irys, and 27.6% in cv. Sárpo Mira. No significant differences in CesA4 localization between mock-inoculated cv. Irys (mean number of gold particles = 90) and cv. Sárpo Mira (mean number of gold particles = 87) were observed (Figure 7A). Decreased deposition of CesA4 in PVY infected plants of both cultivars was statistically valid and was greater in cv. Sárpo Mira (HR) than in cv. Irys (compatible). The CesA4 antigen was detected primarily in membranous structures and organelles, also in the TGN and ER, in both mock-inoculated and inoculated potato plants (Figure 8B). Statistical analyses of CesA4 antigen concentration showed that significant decline in levels of CesA4 accumulation occurred in PVY-infected compared to mock-inoculated potato plants (Figure 8B). High amounts of CesA4 antigen were detected in the plasma membrane and cell wall in both cultivars. ANOVA evaluations of CesA4 antigen levels showed statistically significant differences between localization patterns in relation to PVY resistance level. In resistant plants, similar CesA4 levels founded within both vacuole and plasma membrane and were noticed in lower levels than in mock-inoculated and infected susceptible plants. In susceptible cultivars, levels of CeA4 antigen in plasma membrane, cell wall and ER were higher.

These data indicate that CesA4 synthesis reduction is greater during viral infection in cv. Sárpo Mira than cv. Irys plants. HR reaction induced by PVY (cv. Sárpo Mira) caused high accumulation of CesA4 inside the vacuole. Such high levels of CesA4 deposition in vacuole were observed neither in mock-inoculated nor infected susceptible plants.

### 2.4. Localization of Extensin (HRGPs) in Susceptible and Resistant Potato-PVY^NTN^ Interactions

**Figure 9:**
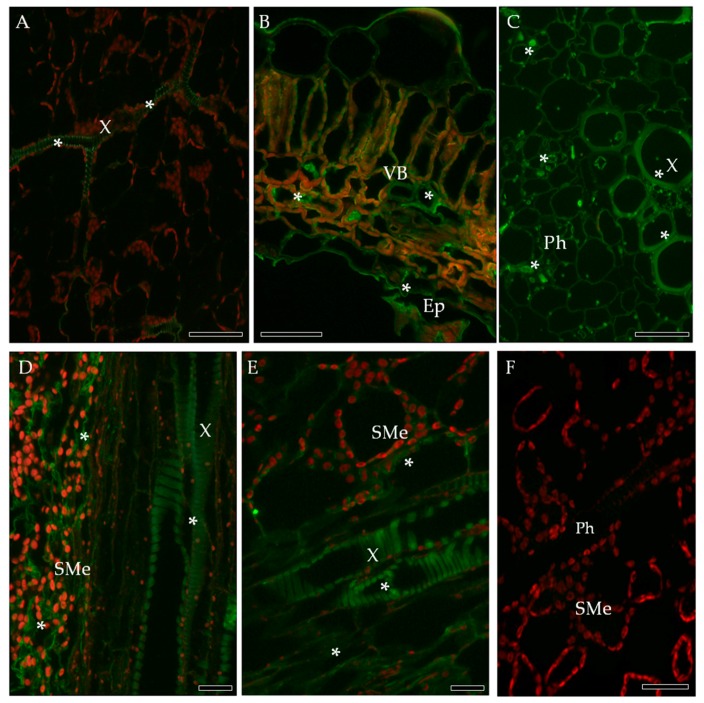
Fluorescence detection of extensin (HRGP) in potato–PVY^NTN^ compatible (**B**,**C**) and incompatible interaction (**D**–**E**). (**A**) Extensin signal (*) in xylem elements of mock-inoculated leaf. (**B**) Extensin in xylem cell wall, spongy mesophyll symplast as well as in epidermis in Irys leaf cross section inoculated with PVY^NTN^ (asterisk). (**C**) Green fluorescence signal of extension (*) in phloem and xylem elements in Irys leaf blades. (**D**) Fluorescence signal in the cell wall of xylem and spongy mesophyll in HR. (**E**) Fluorescence extension signal in phloem and xylem elements in HR. (**F**) Control—lack of green fluorescence signal in hypersensitive response when primary antibodies were omitted. Bar 200 µm. Ep—epidermis, Ph—phloem, SMe—spongy mesophyll, VB—vascular bundle, X—xylem.

**Figure 10:**
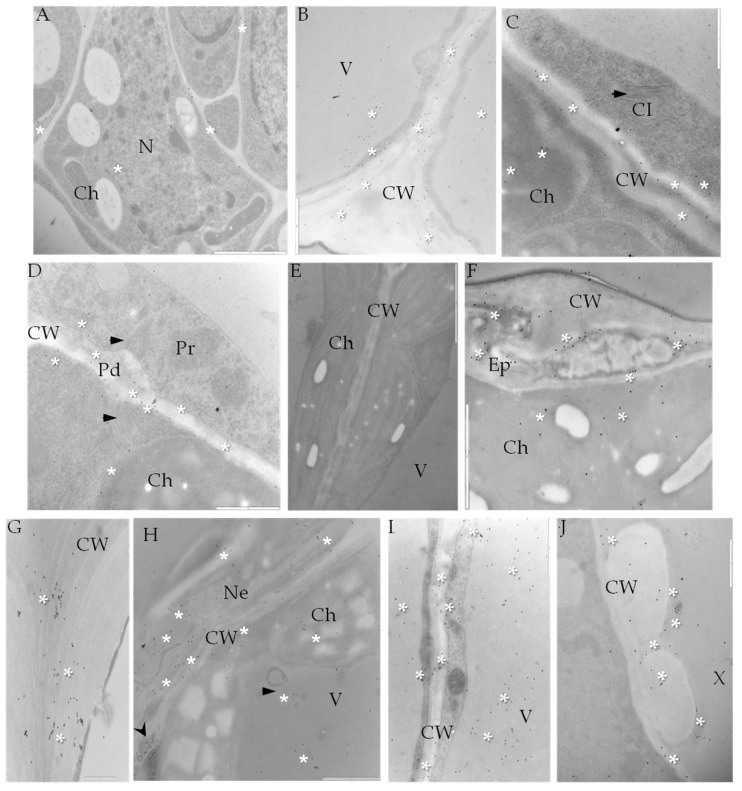
Immunogold labeling of extensin (HRGP) in potato–PVY^NTN^ compatible (**B**–**D**) and incompatible interaction (**E**–**J**). (**A**) Gold deposition (*) of extensin in phloem parenchyma cells of mock-inoculated potato. Bar 1 µm. (**B**) Extensin localization (*) in epidermis cell wall and vacuole. Bar 1 µm. (**C**) Extensin localization (*) in cell wall and chloroplast in mesophyll cell of potato Irys. Virus cytoplasmic inclusion (arrow) in cytoplasm. Bar 1 µm. (**D**) Extensin deposition (*) around plasmodesmta associated with virus cytoplasmic inclusion (arrow). Bar 1 µm. (**E**) Control—lack of gold deposition in hypersensitive response when primary antibodies were omitted. Bar 2 µm. (**F**) Extensin gold deposition (*) in necrotized epidermis and inside chloroplast in Sárpo Mira mesophyll cell. Bar 3 µm. (**G**) Extensin deposition (*) in epidermis cell wall in Sárpo Mira. Bar 1 µm. (**H**) Extensin localization (*) in necrotized mesophyll area in hypersensitive reaction. Deposition also around multivesicular bodies (arrow) and paramural bodies (arrowhead). Bar 2 µm. (**I**) Extensin deposition (*) in cell wall, cytoplasm and vacuole of mesophyll cell in potato Sárpo Mira. Bar 2 µm. (**J**) Extensin localization (*) in xylem tracheary elements. Bar 1 µm. Ch—chloroplast, CI—cytoplasmic inclusions, CW—cell wall, Ep—epidermis, ER—endoplasmic reticulum, M—mitochondria, N—nucleus, Pd—plasmodesmata, Pr—peroxisome, SE—sieve element, V—vacuole, VP—virus particles, X—xylem tracheary element.

Localization of extensin (hydroxyproline-rich glycoproteins, HRGPs), in the PVY^NTN^-infected susceptible (Irys) and resistant (Sárpo Mira) potato cultivars was investigated by immunofluorescence. In compatible interaction, the strong green fluorescence signal of extensin was observed on the surface of epidermial cell walls as well as in both xylem and phloem tissues (Figure 9B,C) in comparison to controls (Figure 9A,F). In the resistant cultivar (HR), HRGPs antigen occurred predominantly in xylem and phloem elements, and was also visible in spongy mesophyll cells (Figure 9D,E). In cv. Irys (compatible interaction) extensin was detected by immunogold labeling in the cell wall area, but primarily in symplast of epidermis (Figure 10B) and mesophyll cells, especially when virus particles or inclusions were deposited in cytoplasm (Figure 10C,D). In the incompatible interaction (HR) the level of HRGPs deposition was visibly greater (Figure 10F–J). The localization occurred in lower and upper epidermis, and also in necrotic areas (Figure 10F,G), mesophyll (Figure 10H,) as well as in xylem tracheary elements (Figure 10J).

**Figure 11:**
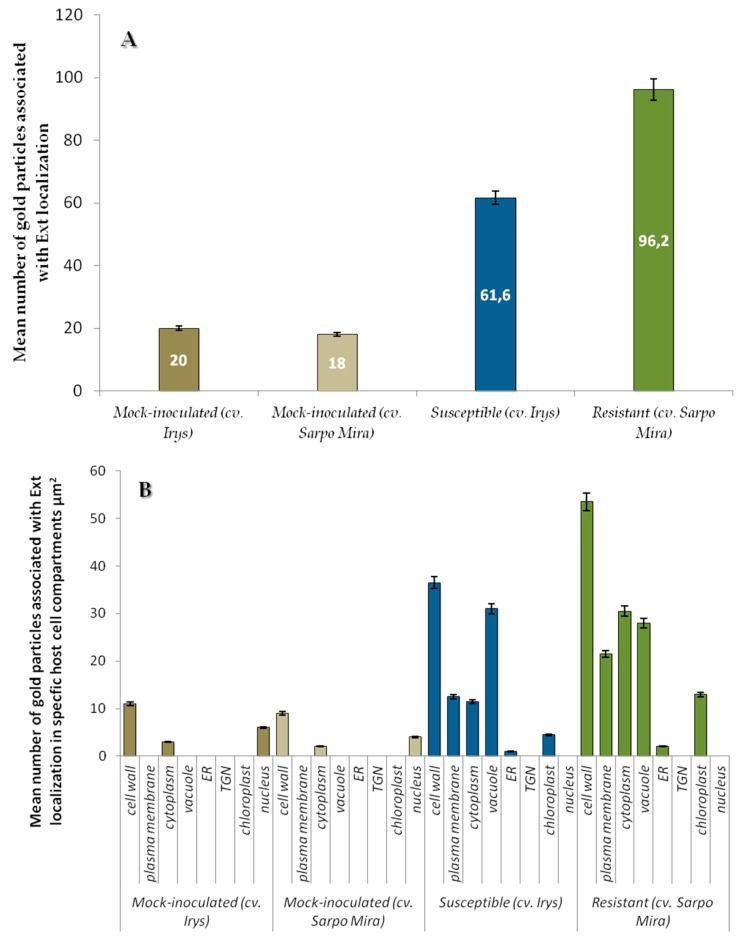
Statistical significance assessment of extensin (HRGP) epitopes immunogold localization (**A**) in mock-inoculated and PVY inoculated potato tissues of cultivars Irys (susceptible) and Sárpo Mira (resistant) & (**B**) in specific host cell compartments of mock-inoculated and PVY inoculated potato plants (susceptible and resistant). Figure presents mean numbers of gold particles located in specific compartment per µm^2^. Quantification immunogold localization was prepared using ANOVA method. Mean values of gold particle localization were evaluated at the *p* < 0.05 level of significance using post-hoc Tukey HSD test.

Quantification by immunogold localization of extensin epitopes revealed an increase of HRGPs in potato plants infected by PVY^NTN^. The mean number of gold particles was 61.6 for the compatible interaction and 96.2 for the HR interaction (Figure 11A). The level of detectable extensin in mock-inoculated cv. Sárpo Mira plants was estimated as lower than in cv. Irys. Therefore, the induction of extensin by PVY^NTN^ infection in different cultivars was at various level of intensity. Analyzed data showed that induction of extensin in resistant plants during PVY infection was more rapid and intense. Further analyses of variances in specific cell compartments indicated more interesting results. In all type of controls we neither observed localization of extensin in cell membrane and vacuole, nor in TGN and ER (Figure 10A,E and Figure 11B). Regardless of cultivar resistant level, PVY infection induced presence of extensin in plasma membrane, vacuole or ER in contrast to mock-inoculated plants. The highest level of extensin was detected in resistant cultivars (Figure 11B), with the highest levels in cell wall and cytoplasm, and the lowest in ER. Interestingly, the localization of extensin in vacuoles was significantly greater in infected cv. Irys than in cv. Sárpo Mira. In infected susceptible potato cv. Irys localization pattern was different from observed in cv. Sárpo Mira. Extensin accumulation was largest in the cell wall and vacuole and least in the ER in the compatible interaction. The statistical data indicate that viral infection induced different patterns of extensin localization within potato cell compartments depending on plant-host interaction. It appears possible that in resistant potato cultivars, HRGPs translocation is diverted from vacuoles to cell walls. In contrast, in susceptible cv. Irys plant-PVY^NTN^ interaction extensin accumulation occurred in the vacuole. These data show clearly that location of extensin in the cell wall during HR is crucial for cell wall modification, which is a part of the complex system providing resistance to PVY infection.

## 3. Discussion

Successful systemic infection of the plant host by plant virus infection depends on its ability to overcome the several barriers to cell-to-cell transport. After cell entry via the host symplast and initiation of replication, systemic infection is successful when progeny virus is able to move freely to adjacent cells. Plasmodesmata act as intercellular connections between plant cells [18] with the help of the host cytoskeleton and endomembranes, which can facilitate virus symplastic movement [19]. The plant cell wall is the first contact area, which plays an important role in initiation as well as regulation processes of defense response. A deeper understanding of the different properties and activities of the plant cell wall is of fundamental importance for understanding the evolution of plant defense strategies. A number of recent publications have proposed models of host–plant cell wall modifications in response to actively penetrating pathogens such as fungi, nematodes and bacteria. Interaction between plant pathogens and the host cell wall depend on the lifestyle of the pathogen. For example, necrotrophs kill cells and macerate dead plant tissues by secreting enzymes, which are able to degrade the cell wall [20]. Bio- and hemi-biotrophic pathogens are dependent on living tissues, and therefore require a different strategy for interaction with the cell wall. Cell wall penetration by fungi or oomycetes utilizing haustoria as feeding structures involves minimum cell wall damage, whereas bacteria potentially delivers effector proteins to the cell wall [21]. Cell wall associated defense mechanisms are designed to retard disease development at an early stage or even to eliminate the pathogen as in the case of a hypersensitive response. In potato two types of resistance genes to PVY have been identified, *Ry* causing symptomless extreme resistance (ER) and *Ny* inducing a hypersensitive response (HR). The potato cv. Sárpo Mira originating in Hungary revealed a very high resistance level to PVY, described as score 9 (in a 1–9 scale accordingly to the European Cultivated Potato Database) [22]. Plants reacted with hypersensitive necrosis after PVY^NTN^ inoculation in detached leaves and the whole plant [23]. 

Two classes of plant responses to pathogens are currently recognized. The first is the pathogen or microbe associated molecular pattern (PAMP or MAMP) in which plant recognition receptors (PRR) form PAMP- or MAMP-triggered immune responses (described as PTI) [24]. The second class involves intercellular receptors of pathogen virulence called effectors (effector-triggered immunity or ETI) [25]. Viruses do not code for PAMPs or ETIs and antiviral immune responses are triggered by the resistance proteins, which are not yet classified as ETI. At the molecular level many cascades of genetic signaling could be activated in HR to induce expression of defense proteins. Primary signaling cascades are associated with expression or even up-regulation of several defense proteins such as glucanases, chitinases, and other pathogenesis-related proteins [7,26]. Commonly known host responses involved in HR include ion fluxes, oxidative burst-production reactive oxygen species, localized cell death at site of pathogen infection or cross linking of the cell wall proteins [27,28]. Our previous studies determining the hypersensitive response in potato cv. Rywal described ultrastructural alterations with various organelles changes [29,30], but precise ultrastructural analyses of apoplast area rearrangements in PVY infection are still lacking. Our paper concentrated on PVY^NTN^ impact on cell wall ultrastructure modification and localization of proteins associated with structure and/or modification of the cell wall. In a compatible potato-PVY^NTN^ interaction loss of cell wall structure, association of virus cytoplasmic inclusions with plasmodesmata and active formation of paramural bodies (PMB) were observed. It has previously been reported that the viral movement protein (MP) is able to modify the plasmodesmata by changing the size exclusion limit (SEL) [31]. Despite movement proteins in plant-virus interaction, viruses require also some host factors to support movement process. However, the connections between plasmodesmata desmotubule and potyvirus inclusions with associations of vesicular structure and strong deposition of P3N-PIPO protein could be postulated as cell-to-cell translocation complex machinery [32]. Cytoplasmic inclusions (CI) were directed to plasmodesmata through interaction with P3N-PIPO. An important feature of plant response to pathogen infection is the alteration of the host cell walls which leads to the formation of paramural bodies [33] in the form of multivesicular compartments, but usually associated with cell wall and attached to plasma membrane [34]. The role of vesicular structure in virus-associated ultrastructural modifications has been described for *Potato virus M* [35]. These modifications include the formation of vesicles and tubules originating from the border of the cell wall and intruding into the plasmalemma.

In the PVY^NTN^-Sárpo Mira hypersensitive reaction cell wall disruption (invaginations with folding) and thickening were mainly observed. Additionally, typical for potato-PVY^NTN^ HR interaction ultrastructural changes consisted of phenolic compounds in the apoplast and vacuole. Electron dense deposits within and between the wall and plasmolemma were usually found in mesophyll and epidermis. Callose-like deposits were observed around sieve plates and cell walls primarily in phloem cells. According to Chappell et al. [36], the apoplast is the source of, or the route for, signals related to defense response. Induction of phenolic compound accumlation in cell walls may serve as a physical barrier to invading pathogens [37]. In host-plant reaction to infection by powdery mildew, *Blumeria graminis* (Bgh) or *Colleotrichum lindemuthianum,* phenolics were distributed throughout the cells and host-localized material were shown at the side of infected cell [33,34,38]. The partial retraction of the protoplast from the wall, as observed in hypersensitive reaction, especially in mesophyll cells between necrotized and unaffected, was cited, along with the aforementioned phenolic barrier, as an “ultrastructural symptom” of physical separation [6,39]. This phenomenon may possibly limit contact between infected and non-infected cells and may possibly be involved in hypersensitive reaction. Similar to PVM-red kidney bean interaction [35] and PVY^NTN^–potato cv. Rywal interaction [40], the cell wall thickening in hypersensitive reaction PVY^NTN^–potato cv. Sárpo Mira was mainly observed in mesophyll and phloem parenchyma cells. As postulated by O’Brien et al. [41], strengthening of the plant cell wall may play a key role in host-plant defense. Strengthening of the cell wall could be accomplished by deposition of wall material associated with vesicular structure secretion, such as paramural bodies or multivesicular bodies (MVB), as observed in hypersensitive response in Sárpo Mira tissue. These participate either in the degradation process in the vacuole or fuse to the plasma membrane to release their vesicles [42].

Comparative transcriptome profiling and microarray analysis of gene expression in plant virus infection in susceptible as well as resistance hosts indicate that the cell wall could be a kind of target for different plant viruses. It was reported that the cell wall related genes and transcripts are differentially expressed and regulated during the interactions [31,43,44,45]. Proteins/enzymes involved in plant cell wall organization and metabolism participate in the interaction through which they could influence virus spread [46]. Callose in the apoplast area is directly controlled by the proportion between the actions of two proteins: callose synthase and β-1,3, glucanases that hydrolyze callose [47]. Class II β-1,3 glucanases include pathogenesis-related protein PR-2. It is regarded as cellular factor controlling virus movement [48]. It has also been postulated [49] that TMV infection in tobacco increases the activity of PR-2, which facilitates virus movement. Suppression of β-1,3 glucanase results in increased callose deposition in the cell walls, reducing plasmodesmatal size exclusion limit. This process also restricts short and long-distance transport of PVX (*Potato virus X*), TMV (*Tobacco mosaic virus*) and TNV (*Tobacco necrosis virus*). Alternatively, overexpression not only of β-1,3 glucanase (class II) but also class III glucanase in PVY^NTN^ infection expedited virus spread between cells [46,50]. Our results correspond with analysis of PR-2 in TMV infection. In incompatible interaction we observed callose by ultrastructural analyses. Immunolocalization analyses revealed lower deposition of PR-2 in the hypersensitive than in the compatible interaction. It is generally regarded that glucanases may potentially be localized preferably in vacuole and cell wall [51]. This was confirmed by our results, independently on the type of the interaction, cell wall and vacuole deposition of PR-2 dominated, but also cytoplasm localization was noticed, it indicated probably, that transport of PR-2 in vesicles and membranous structure are possible. Similar phenomena suggested Epel [52]. Furthermore, resistant tobacco plants (VAM) infected with PVY reacted by accumulation of transcript connected with PR proteins [53]. It was summarized that pathogenesis related genes were almost always up-regulated at later stage of infection (late than 2 days after infection). Respectively, in hypersensitive response Sárpo Mira potato plant-PVY^NTN^ interaction, the induction of PR-2 deposition was noticed compared to mock-inoculated control plants. Additionally, also the transcriptome analysis followed by Babler et al. [54] recognized potentiall component of plants responses to virus infection, highlighted that, pathogenesis related PR enzymes involved in cell wall organization were upregulated in potato cv. Rywal hypersensitive to PVY inoculation (starting from 1 day after inoculation). On the other hand, for absolutely different plant virus member of the genus *Phytoreovirus* with quite different range of the host *Rice dwarf virus* (RDV) presented potentially similar reaction [44]. Microarray analyses revealed that about 9 days after inoculation of RDV almost all pathogenesis related protein genes expression was enhanced on rice plants as an example of compatible interaction.

This is the first study on the role cellulose synthase subunit 4 (CesA4) in cell wall structural protein modification in PVY^NTN^-host compatible and incompatible interaction. The β-1,4 glucan (cellulose) is produced at the plasma membrane by large glucan synthase complexes referred to as cellulase synthases CesAs. The proposed model for cellulose synthase complex (CSC) synthesis is that it consists of 36 CESA proteins that produces a cellulose fibril containing 36 glucan chains [55,56]. CESA4 as an element of catalytic subunit of cellulose synthase terminal complex, participated in major mechanisms of the cell wall formation, and is required for β-1,4 glucan microfibril crystallization [57]. It functions in cellulose synthesis during secondary cell wall formation with CESA7 and 8 and is required for xylem cell wall thickening [58,59]. Analyses of localization CesA4 in the HR Sárpo Mir-PVY^NTN^ interaction as well as in the susceptible Irys-PVY^NTN^ interaction revealed a decrease in deposition of CesA4 compared to mock-inoculated controls, based on measurement of relative protein levels detected by immunoassay. These results appear to be compatible with those reported by Humphrey et al. [60] stating that reduction in cellulose content is a feedback response associated with biotic as well as abiotic stress. Hernandez-Blanco et al. [59] postulated that the production of CESA4 mutants 7 or 8 is triggered in response to resistance to two types of pathogenic bacteria and fungi. The above data is supported by research conducted by Burton et al. [61], showing that the putative cellulose synthase gene (*Cesa*) induced gene silencing when inserted into a *Potato virus X* vector in *N. benthamiana*, resulting in a 25% decrease in cellulose content in leaves tissue. From this it was concluded that phenotypic changes, for example changes in cell wall composition, in infected leaves were associated with reduced levels of CesA mRNA [61]. In contrast, transcriptome analyses during rice response to *Rice stripe virus* infection gave ambiguous results. The cellulose synthase genes after virus inoculation were markedly up-regulated in the resistant host but were down-regulated or even unchanged in the susceptible rice cultivar [43]. Interestingly, the microarray analysis conducted starting from 12 days after RSV inoculation revealed cellulose synthase significantly down-regulated. Similarly, in an investigation conducted by Shimizu et al. [44] for *Rice dwarf virus* 9 days after inoculation, there was significant repression of the genes for proteins generally involved in cell wall synthesis. The key enzyme involved in cell wall synthesis and remodeling, cellulose synthase, was suppressed, but this was mainly observed for the compatible interaction. Despite the reduction in immunodetection of CesA4 deposition in resistant and susceptible plants, the localization pattern strongly indicated that plasma membranes and vacuolated membranous structures, rather than cell walls, were the preferable areas for CeaA4 location independent of host resistance. These findings would be compatible with the assumption that cell walls are composed mostly of polysaccharides and are mainly produced in the Golgi, then secreted to the apoplast where they could be modified or join to the growing cell wall [62,63]. It was ascertained that the β-1,4 glucan cellulose is made on the plasma membrane by CesA complexes, which are assembled in the endoplasmic reticulum or Golgi network [15,64]. The process of delivery and endocytosis of the cellulose synthase complex to and from the plasma membrane are essential for regulation of cellulose biosynthesis, also in stress response [56]. This trafficking pathway could be the explanation for increased deposition of CesA4 proteins in the vacuole in HR reaction, and the relatively lower rate of CesA4 protein in the resistant plant. Vacuolar localization may be an effect of sequestration of CesA4 to the vacuole by an active participation of multivesicular bodies or prevacuolar vesicles. These findings suggest that inhibition of CesA4 deposition as a consequence of PVY^NTN^ leads to generate antimicrobial process associated with resistance to the pathogen. This inhibition could be an effect of sequestration of CesA4 to the vacuole thus vesicular structures, in response to HR reaction.

Based on analyses of the localization of extensin (hydroxyproline-rich glycoprotein, HRGP) involved in cell wall structure and modification, it may be concluded that HRGP deposition is induced in both susceptible and resistant cultivars by PVY^NTN^ infection, however, the post-inoculation levels were much higher in the hypersensitive than in the compatible host-pathogen interaction. HRGP epitopes (detected by LM1 antibodies) were located especially in both vascular tissues, moreover, preferably in apoplast region, but in symplast the deposition was also noticed with the special consideration of vacuole, membranous structures and cytoplasm. Our observations and analyses are consistent with data presented by O’Connel et al. [65], who postulated that HRGP protein may be localized not only in the cell wall but is concentrated close to plasma membrane and membranous structures in bean tissues infected with *P. syringae* as well as *Colletotrichum*. Our analyses are consistent with the findings that TMV infection acts as a biotic factor inducing HRGP in tobacco [66]. Similarly, Wycoff et al. [67] examined transgenic tobacco, containing a HRGP-GUS gene and inoculation also with TMV. The authors reported that 27 test plants had an increase of 2.5-fold GUS activity compared to mock-inoculated plants. In our experiment HRGP epitopes were mainly localized in both vascular tissues or even in epidermis as reported by Templeton et al. [68], whose in situ hybridization analyses found accumulation of HRGPs transcripts in the epidermis and in the outer phloem in the vascular area. It was postulated that HRGPs accumulate as a soluble cell wall protein at main stages of development and could be made insoluble by intermolecular cross-linkage formation [69]. According to Raggi [70], the activation by PVY^N^ infection of HRGPs in the cell wall of tobacco leaves and resultant cross -linking and cell wall lignifications contributes to resistance against *Erysiphe cichoracearum* by reduction in fungal haustoria production. These cross-linking process has been proposed as a mechanism of strengthening the cell wall [71]. Biotic stress induced HRGP (extensin) provides a structural barrier against the pathogen [66,72]. Based on our findings and postulated literature information, the induction of extension (HRGP) in hypersensitive Sárpo Mira clearly indicated that we observed reinforcement of the cell wall and active distribution of HRGP, which are important in preventing the entry-point of PVY in leaf tissues. Moreover, the deep sequencing analysis describing host response to *Rice stripe virus* infection indicated that cell wall strengthening was induced especially in resistance rice cultivars by markedly up-regulation glycine-rich repeat proteins [43]. 

## 4. Materials and Methods

### 4.1. Plant Material and Virus Inoculation

*Solanum tuberosum* L. cvs. Irys (PVY^NTN^ resistance score 5.5 in a 1–9 scale) [73] and Sárpo Mira (resistance score 9) [22] were obtained from IHAR-PIB, Plant Breeding and Acclimatization Institute, Bonin Research Center. Plants were grown at 20 °C, under 16/8-h light/dark photoperiod and a light intensity of 400 μmol m^−2^ s^−1^, and were inoculated mechanically as previously described [74] at the four leaf stage with the NTN strain of PVY. Potato cv. Sárpo Mira developed a hypersensitive necrotic response visible at 7 days post-inoculation. This reaction is conferred by the *Ny-Smira* gene located on the long arm of the potato IX chromosome [23]. Hypersensitive reaction symptoms on inoculated leaves appeared 7 days post-inoculation. Potato cv. Irys reacted with systemic necrosis visible at 10 days post-inoculation. Leaves from both PVY^NTN^-infected and healthy controls inoculated with phosphate buffer were collected 10 days post-inoculation and tested by DAS-ELISA [75].

### 4.2. Ultrastructural Examinations

Potato (infected and mock-inoculated) leaf samples (2 mm × 2 mm sections) were fixed in 2% (*v*/*v*) paraformaldehyde and 2% (*v*/*v*) glutharaldehyde in 0.1 M cacodylate buffer (pH 7.2) for 2 h and washed 4 times in cacodylate buffer [30]. Samples were post-fixed in 2% (*v*/*v*) OsO_4_ for 2 h at 4 °C, dehydrated in an ethanol series (10–99%) and propylene oxide, embedded in epoxy resin (EPON, Fluka, Switzerland) and polymerized at 60 °C for 24 h. Ultra-thin (90 nm) sections were stained with uranyl acetate/lead citrate (Sigma-Aldrich, St. Louis, MO, USA) and examined by transmission electron microscopy [76].

### 4.3. Immunofluorescence Localization

Rat IgG monoclonal LM1 antibodies (HRGP/extensin specific) were purchased from PlantProbes (Leeds, UK). Polyclonal rabbit anti-CesA4 and anti PR-2 antibodies were obtained from Agrisera (Vӓnӓs, Sweden). Potato leaf tissue samples were fixed in 4% (*w*/*v*) paraformaldehyde in 0.1 M microtubule stabilizing buffer (MSB) pH 6.9 containing 0.1% (*v*/*v*) Triton X-100 for 2 h at room temperature as described by Gubler [77]. Samples were dehydrated in an ethanol series containing 10 mM dithiothreitol and embedded in butyl-methyl-methacrylate (BMM) resin and polymerized under UV radiation for 20 h at −20 °C. Acetone was used to remove the BMM from 2 μm sections collected on silane slides (Thermo-Fischer Scientific, Warsaw, Poland). Immunofluorescence analysis was carried out after pre-incubation in 3% (*w*/*v*) bovine serum albumin in PBS for 1 h at room temperature. Sections on slides were incubated for 2 h in a humid chamber with antibodies diluted in PBS (1:50 *v*/*v* for polyclonal and 1:10 *v*/*v* for monoclonal). Controls consisted of mock-inoculated tissue and pre-immune serum. After rinsing with PBS-Tween20 buffer slides were treated with secondary goat anti-rabbit IgG conjugated with FITC (fluorescein isothiocyanate) goat anti-rat IgG in PBS at RT in the dark for 2 h. An Olympus AX70 Provis (Olympus Poland, Warsaw, Poland) with a UM61002 filter set and equipped with an Olympus SC35 camera was used for fluorescence imaging [30,74].

### 4.4. Immunogold Labeling

Leaf sections (50–70 nm thick) were mounted on Formvar-coated nickel grids and treated with 10% hydrogen peroxide solution for 10 min to remove the resin. The grids were pre-incubated for 1 h in blocking medium containing 2% normal goat serum (Thermo-Fischer Scientific) with 3% BSA in 0.1 M PBS buffer (pH 7.6) as described previously by Otulak and Garbaczewska [76]. Grids were then rinsed three times in buffer containing 0.05% Tween-20 for 10 min and treated for 2 h at room temperature with primary antibodies (as mentioned in above section) in PBS and washed in PBS-Tween 20. The samples were treated for 1 h with gold-conjugated secondary antibody (15 nm, Sigma-Aldrich, Warsaw, Poland) and then rinsed first in PBS and then in distilled water for 5 min. Labeling specificity was checked by incubating grids with material from mock-inoculated plants and by omission of the primary antibody from incubating solution. The grids were then counterstained with 1% uranyl acetate for 5 min and washed 5 × 2 min with distilled water. Immunogold-labeled sections on grids were examined by transmission electron microscope (as described above).

### 4.5. Statistical Quantification of Tissue and Cell Distribution of Immunogold-Labeled Cell Wall-Associated Proteins PR-2, CesA4 and HRGP (Extensin)

Labeling quantification of cell wall associated proteins PR-2, CesA4 and extensin was based on the method proposed by Luschin-Ebengreuth and Zechmann [78], with modifications of the type of statistical method and program used for statistical analyses. Data on gold particle concentration was investigated by analysis of variance (ANOVA) and the post-hoc Tukey HSD test in STATISTICA software (StataSoft and TIBCO Software Inc., Palo Alto, CA, USA, version 13.0). ANOVA was used as an efficient estimator of gold labeling. Each cell wall-associated protein was investigated individually. For statistical estimation of immunogold labeling we compared infected and healthy (mock-inoculated) plants from different cultivars. Gold particles in tissues/cell compartments were counted in forty 10 μm^2^ fields per image. In each combination (two mock-inoculated, cv. Irys and cv. Sárpo Mira PVY^NTN^ inoculated potato) gold particles from 200 photos were counted for PR-2, CesA4 and HRGP. Data were based on analyses of PR-2 (600 photos), CesA4 (600 photos) and HRGP (600 photos).

## 5. Conclusions

Recently, transcriptome analysis research on responses triggered during plant-pathogen interactions have increased. These investigations delivered new findings relating to up- and/or down-regulation of genes involved in pathogen infection. This study utilizes ultrastructural studies and immunodetection of specific proteins to obtain a better understanding of the involvement of cell wall components in response to biotic stress. The ultrastructural analyses and precise localization and distribution of cell wall proteins/enzymes permitted the collection in situ of new information on structural changes, reorganization and remodeling within the apoplast and symplast associated with plant-host defense response to virus infection. Previous research has concentrated on cell wall changes in different plant-pathogen interaction models of plant pathogenic bacteria or fungal, but not virus-host-plant interactions. In contrast to bacterial and fungal pathogens, viruses are not mechanical destructors of host cell walls. Our studies for the first time have precisely described the ultrastructural changes accompanying the two types of PVY^NTN^-potato interactions, compatible and incompatible. Our findings revealed, that deposition of proteins (e.g., CesA4) involved in potato cell wall synthesis was suppressed 10 days after PVY inoculation in compatible as well as hypersensitive reaction. In contrast, we observed, in inoculated but not in mock-inoculated controls, the induction of proteins (e.g., PR2 and HRGP) involved in cell wall modification processes. Additionally, the deposition and distribution of PR-2 protein was significantly lower in hypersensitive cultivar Sárpo Mira than in the compatible interaction, and as a consequence the deposition of callose-like materials was observed more frequently. In contrast, HRGP (extensin) localization as measured by cell wall thickening was significantly greater in the HR than the compatible reaction. From these observations it may be concluded that cell wall modifications as determined by protein analysis as well as the symplast are involved in apoplast changes in both types of virus-host interaction. The results of the study on cell wall alterations in PVY^NTN^-resistant and susceptible interaction have paved the way for subsequent studies focused on the potential involvement of other proteins/enzymes and their functions in defense response. Further molecular and cellular studies are needed to elucidate the possible role of other cell wall functions (e.g., expansins and xyloglucan metabolism and distribution) that are potentially active in PVY-host-plant interactions.
